# Tumor Segmentation and Feature Extraction from Whole-Body FDG-PET/CT Using Cascaded 2D and 3D Convolutional Neural Networks

**DOI:** 10.1007/s10278-020-00341-1

**Published:** 2020-05-06

**Authors:** Skander Jemaa, Jill Fredrickson, Richard A. D. Carano, Tina Nielsen, Alex de Crespigny, Thomas Bengtsson

**Affiliations:** 1 Genentech, Inc., South San Francisco, CA USA; 2 F. Hoffman-La Roche Ltd., Basel, Switzerland

**Keywords:** FDG-PET, Deep learning, Lymphoma, DLBCL, NHL, Tumor segmentation

## Abstract

^18^F-Fluorodeoxyglucose-positron emission tomography (FDG-PET) is commonly used in clinical practice and clinical drug development to identify and quantify metabolically active tumors. Manual or computer-assisted tumor segmentation in FDG-PET images is a common way to assess tumor burden, such approaches are both labor intensive and may suffer from high inter-reader variability. We propose an end-to-end method leveraging 2D and 3D convolutional neural networks to rapidly identify and segment tumors and to extract metabolic information in eyes to thighs (whole body) FDG-PET/CT scans. The developed architecture is computationally efficient and devised to accommodate the size of whole-body scans, the extreme imbalance between tumor burden and the volume of healthy tissue, and the heterogeneous nature of the input images. Our dataset consists of a total of 3664 eyes to thighs FDG-PET/CT scans, from multi-site clinical trials in patients with non-Hodgkin’s lymphoma (NHL) and advanced non-small cell lung cancer (NSCLC). Tumors were segmented and reviewed by board-certified radiologists. We report a mean 3D Dice score of 88.6% on an NHL hold-out set of 1124 scans and a 93% sensitivity on 274 NSCLC hold-out scans. The method is a potential tool for radiologists to rapidly assess eyes to thighs FDG-avid tumor burden.

## Introduction

^18^F-fluorodeoxyglucose positron-emission tomography (FDG-PET) is a widely used imaging modality in oncology, where radiolabeled glucose is intravenously administered and is rapidly taken up by metabolically active tumors. This imaging technology provides a means to visualize and quantify metabolically active tumor burden in patients, and FDG-PET has been applied to a wide range of cancer types, with differing degrees of FDG uptake. Some tumors (e.g., prostate cancer) exhibit relatively low FDG uptake and, thus, may not be detectable by FDG-PET, whereas many other tumor types (e.g., non-small cell lung cancer, non-Hodgkin’s lymphoma) demonstrate high FDG uptake, making them highly visible in FDG-PET images [1]. FDG-PET has been found to be superior to anatomical imaging modalities (cf., MRI, CT, US) for detection of these FDG-avid tumors [1] and FDG-PET tumor burden metrics [2] have been shown to be prognostic of clinical outcome [1, 3], Moreover, FDG-PET imaging may provide an early indicator of therapeutic efficacy and is an established modality in the assessment of response to treatment in patients with malignant lymphomas [4–6].

Analysis and interpretation of FDG-PET images is performed by trained radiologists or readers who visually inspect the images for tumors and then define individual tumor boundaries (region of interest, ROI) manually, or with the use of semi-automated image analysis software. Typically, the maximum standardized uptake value (SUV) within a tumor ROI is recorded along with the tumor volume and tracked over the course of treatment. Manually based analyses can be very labor-intensive and time-consuming, especially in whole-body FDG-PET scans. Additionally, manually driven analyses will suffer from intra- and inter-reader variability.

The development of a fully automatic segmentation algorithm, which aims to increase both speed and reproducibility of scan assessments, faces significant technical challenges. For instance, specific FDG uptake occurs in a number of highly metabolic but normal, healthy tissues (e.g., brain and heart) and intravenous administration of FDG also produces a time-dependent (relative to time of injection) blood pool signal along with a strong FDG signal in the liver, kidney, and bladder due to the accumulation of the contrast agent in these organs. Thus, any automatic algorithm would need to be able to distinguish normal high uptake versus the accumulation of FDG in tumors that range in value from low to high. Moreover, the volume of FDG-avid tumors is relatively small compared with the volume of non-tumor, FDG-positive regions, resulting in a sparse signal for an image segmentation algorithm to reliably extract. Development of a robust image segmentation algorithm faces a further challenge in the high degree of biological intra- and inter-tumor heterogeneity associated with tumor structure, perfusion, and metabolism leading to variability in FDG uptake. In addition, although attempts to standardize imaging protocols have improved acquisition consistency, variability between scans and sites still exists and contribute to the overall variability in the data.

The use and accuracy of convolutional neural networks (CNN) for image segmentation have increased over the last few years [7, 8]. The application of CNNs to medical imaging has also recently grown [9, 10]. While most CNN architectures are applied to 2D images, the increased interest in 3D medical images has contributed to the development of 3D CNNs [11]. These 3D CNNs can be used to exploit the 3D spatial properties of the tissue of interest (e.g., local tumor environment) to aid in the segmentation task.

This paper presents a novel end-to-end, cascaded 2D to 3D CNN architecture to robustly and automatically identify and segment tumors in whole-body FDG-PET images. The overall goal is to provide a tool to efficiently and accurately quantify total metabolic tumor burden in oncology patients. Our algorithm employs a computationally efficient architecture devised to accommodate the size of eyes to thighs scans, the extreme imbalance between tumor burden and the volume of healthy tissue, and the heterogeneous nature of the input images. This fully automated image segmentation algorithm has been successfully applied to two different subtypes of NHL: diffuse large B cell lymphoma (DLBCL) and follicular lymphoma (FL).

## Methods

**Fig. 1:**
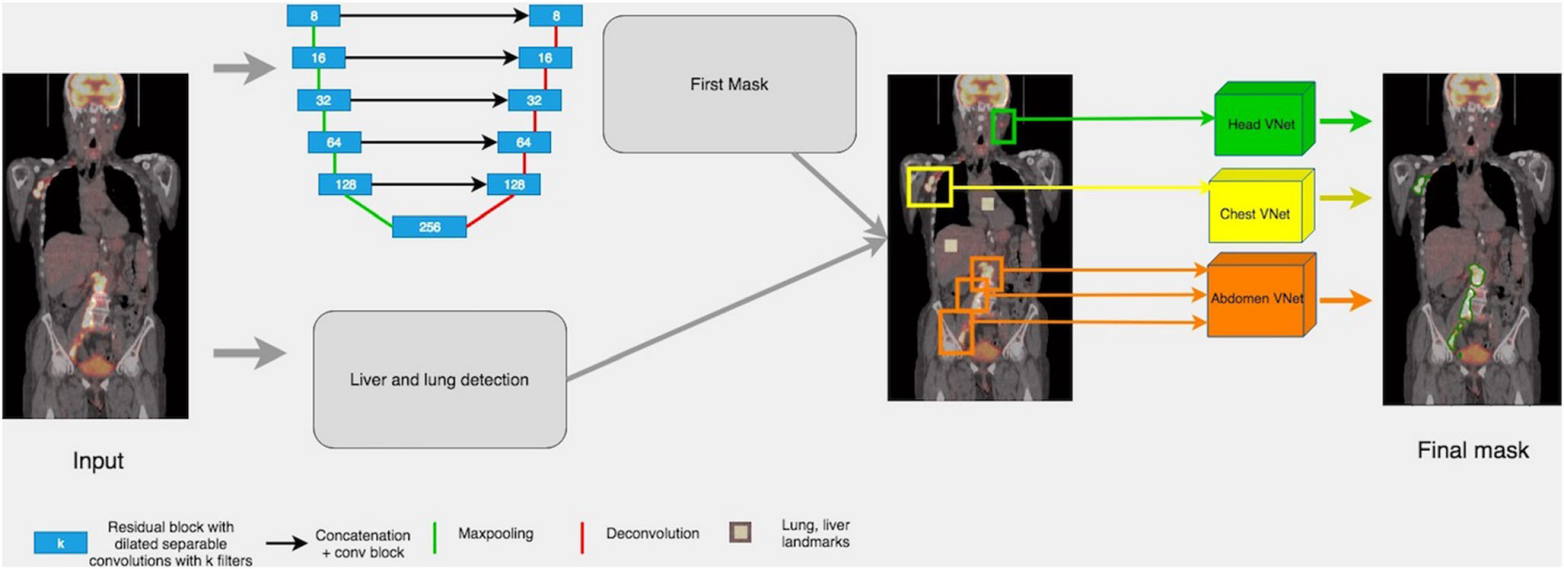
Model architecture. The full pipeline consists of three steps: a 2D segmentation, connected components labeling in three anatomical regions (head-neck, chest, abdomen-pelvis), and a refinement of the 2D prediction using a region-specific 3D segmentation for each region

To automate tumor segmentation, we propose a cascaded 2D and 3D architecture (Fig. 1). This architecture is fast and memory-efficient to deal with the size of the images and adapted to the highly unbalanced nature of the segmentation problem. For good performance, the latter challenge can be addressed with very deep networks; however, deep networks for large inputs are limited by current GPU memory capacity. The presented algorithm addresses these competing challenges by performing 2D axial and sagittal slice-by-slice segmentations, then dividing the body into three different regions and refining the 2D predictions with region-specific 3D CNNs. A multi-term loss and atrous convolutions allows for the detection of small, localized, and diffuse tumors.

### 2D Segmentation

Our first step consists of segmenting image slices individually using a modified U-Net [12]. U-Net has been widely used in segmentation tasks, especially in medical imaging, where skip connections link the global context features learned by the contracting block, while localization are features learned in the expansion block.

**Fig. 2:**
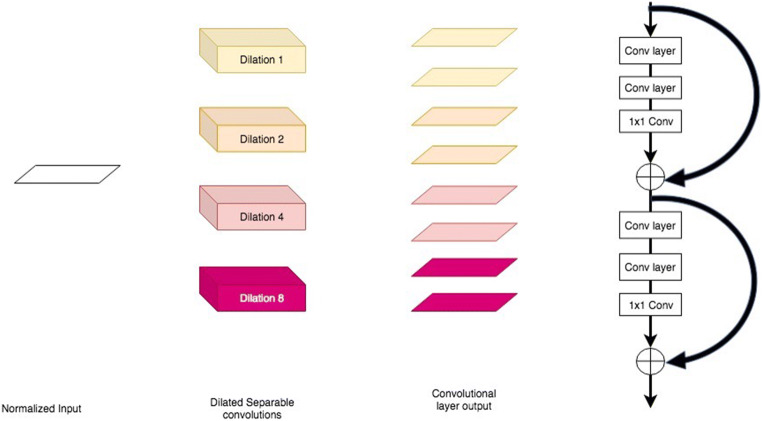
Layer architecture. Our layer contains two residual blocks (on the right). Convolutional layers of the residual block use atrous, separable convolutions at four different scales (on the left). Here, a layer is represented with eight filters

In our architecture, we replace the convolutional blocks composed of two convolutional layers in the original U-Net architecture by two residual blocks [13] with batch normalization and separable convolutions at four levels of dilation (Fig. 2). Empirical evidence [13] shows that residual blocks allow a gain of accuracy and faster optimization. Separable convolutions, depth-wise convolutions followed by point-wise convolutions, have also been shown to provide a large gain in convergence speed and a significant reduction of the model size [14]. Further, dilated convolutions [15] expand the receptive field without loss of resolution, allowing for aggregation of multi-scale contextual information without downsampling. As will be shown, this redesign of the convolutional blocks is effective at extracting very localized and rare information, as typically encountered in FDG-PET/CT scans. Both the FDG-PET and co-localized attenuation-corrected CT images are used as inputs to leverage the structural (CT) and metabolic (FDG-PET) information provided by each modality. The input size is 448 × 512 × 2 for each imaging slice.

### Liver and Lung Detection

CT and FDG-PET images are highly heterogeneous depending on the location in the body due to variability in structure and metabolism across tissues. In order to limit the impact of this variability, we split the body into three anatomical regions: head-neck, chest, and abdomen-pelvis. We automatically assess the location of the liver and the center of mass of the lungs as reference points.

We detect the liver in the FDG-PET data by using an approach similar to a previously published method [16]. We first use the method described in [16] to detect the brain in the FDG-PET images, where the minimal size of the brain was set to 500 mL. The liver is detected in the FDG-PET images by a series of thresholding and morphological operations in the lower left part of the image relative to the brain. First, a threshold of 1.0 SUV, reflecting normal uptake in the liver, is applied to the selected window, followed by a hole filling operation. The binary mask is then eroded by application of a spherical structuring element of radius 8 mm. The liver is identified as the highest connected component with its center of mass in the left third of the sagittal axis of the image.

We follow a similar procedure to [17] to detect the center of mass of the lungs. We threshold the image at – 300 Hounsfield Units (HU) to obtain a binary mask and keep only the 8 largest connected components. In each axial slice, we remove the selected regions adjacent to the slice boundaries, erode the remaining connected components to avoid any leakage, keep only the two largest connected components, and select the center of mass of the 2 largest remaining connected components.

### 3D Segmentation

Given the outputs of the 2D segmentation and depending on their relative location to our references in the liver and chest, we label connected components in the 2D tumor masks. For each of the three anatomical regions, we use a V-Net [11] to refine the 2D segmentation. The network contains four downsampling blocks (16, 32, 64 and 128 filters) and three upsampling blocks. The layers use a ReLU activation and a 3 × 3 × 3 kernel size. We use patches from FDG-PET and CT as a 2-channel input, where the patches are 32 × 32 × 32 × 2 in the head or neck, 64 × 64× 64 × 2 in the chest, and 96 × 96 × 96 × 2 in the abdomen. The different patch sizes were chosen empirically based on investigating the sizes of lesions in each of the regions.

The final mask is obtained by averaging the tumor masks obtained with 2D and 3D segmentations. Experiments on the training set show that averaging the 2 masks produces better results than solely using the 3D masks. Total metabolic tumor volume and the SUV_max_ are derived from these masks.

### Training Loss

In order to deal with the unbalanced nature of the images, where the average proportion of negative voxels in a volume is 99.5% and often higher than 80% in a single slice, we use the Dice Similarity Coefficient (DSC) [18] and a weighted cross-entropy in 2D:

1 $$ L=\left[1-\frac{2\ \left|\ P\cap T\ \right|}{\left|P\right|+\left|T\right|}\right]-\left[{\sum}_{v\in V}\frac{\left|V\right|}{\sum_{v\in V}{y}_v}\left({y}_v\log \left(\hat{y_v}\right)+\left(1-\frac{\left|V\right|}{\sum_{v\in V}{y}_v}\right)\left(1-{y}_v\right)\log \left(1-{\hat{y}}_{\mathrm{v}}\right)\right)\right] $$

In (1), *V* denotes the voxels in an image, *T* the set of positive voxels, *P* refers to the set of predicted positive voxels, *y*_v_ the value of voxel v in the tumor mask, and $\hat{y_{\mathrm{v}}}$ the value of voxel v in the predicted tumor mask.

Similarly, in 3D, we use the DSC, the sensitivity and the mean absolute error in the loss function (2) to minimize the number of false negatives and to avoid the concentration of outputs around 0.5.

2 $$ L=\left[1-\frac{2\ \left|\ P\cap T\ \right|}{\left|P\right|+\left|T\right|}\right]+\left[1-\frac{\ \left|\ P\cap T\ \right|}{\ \left|T\right|}\right]+\left[\frac{\ 1}{\ \left|V\right|}{\sum}_{v\in V}\left|{y}_v-{\hat{y}}_{\mathrm{v}}\right|\right] $$

### Data and Preprocessing

Our complete dataset consists of 3664 eyes to thighs FDG-PET/CT scans collected from multiple imaging sites in three different clinical trials (Goya, *N* = 1418, NCT01287741 [19]; Gallium, *N* = 1401, NCT01332968 [20]; and OAM455g, *N* = 137, NCT00854308). All scans were acquired at baseline and end of treatment with standardized image acquisition protocols and were centrally reviewed by an independent review committee. Each trial had a different independent review committee. This dataset contains scans of 1695 previously untreated patients with Non-Hodgkin’s lymphoma: 1135 diffuse large B cell (DLBCL) and 562 follicular lymphoma (FL) patients. For these scans, radiologist-reviewed annotations of full tumor burden in 3D were available and served as “ground truth.” Additionally, scans from 137 non-small cell lung cancer (NSCLC) patients with annotations of up to five lesions, i.e., “partial ground truth,” were available. Pre-processing steps include overlaying the PET and CT, resampling scans to a constant isotropic voxel size of 2 × 2 × 2 mm, deriving the SUV for PET scans based on information in the DICOM header, and creating coronal and sagittal reformations from the axial acquisitions. Radiologist-derived tumor masks were reconstructed from the available tumor annotation files.

The training dataset consisted of 2266 scans from the DLBCL patients, yielding a total of 861,053 coronal, 770,406 sagittal, and 971,265 axial slices and 13,942 individual tumors. Scans from FL (1124) and NSCLC (274) patients served as the test dataset, approximately a 60:40 split with training data. NSCLC patient scans were excluded from the training set in order to avoid training on data with false negatives. Dividing the data by studies also allows us to test and validate that the model, trained on one cancer type, can be extended to other types of cancer.

### Experiments

Learning rate, kernel size, and network depth were considered for hyper parameter tuning. We varied the learning rate and tested a variable learning rate (cosine annealing) for each network. For 2D CNNs, our experiments included testing 3 × 3 and 5 × 5 kernels. Neither a kernel of 5 × 5 nor an increase in depth from 6 to 7 lead to significant performance gains. We note that almost 90% of the coronal and sagittal slices do not contain tumors; thus, in order to avoid converging to null predictions, we rebalanced the dataset so that approximately 10% of slices did not contain tumors (98,000 training slices).

2D networks were trained on 2 Nvidia Quadro P6000 graphical processing units using the RMSProp optimizer, 25 epochs, and a batch size of 16. We set the learning rate at 10^−5^ for 13 epochs and divided by 2 after every 3 epochs. The V-Nets were trained using the Adam [21] optimizer for 100 epochs with a batch size of 4. The learning rate was set at 10^−4^ for 50 epochs, 10^−4^/2 for 25 epochs, and 10^−4^/4 for 25 epochs.

## Results

**Fig. 3:**
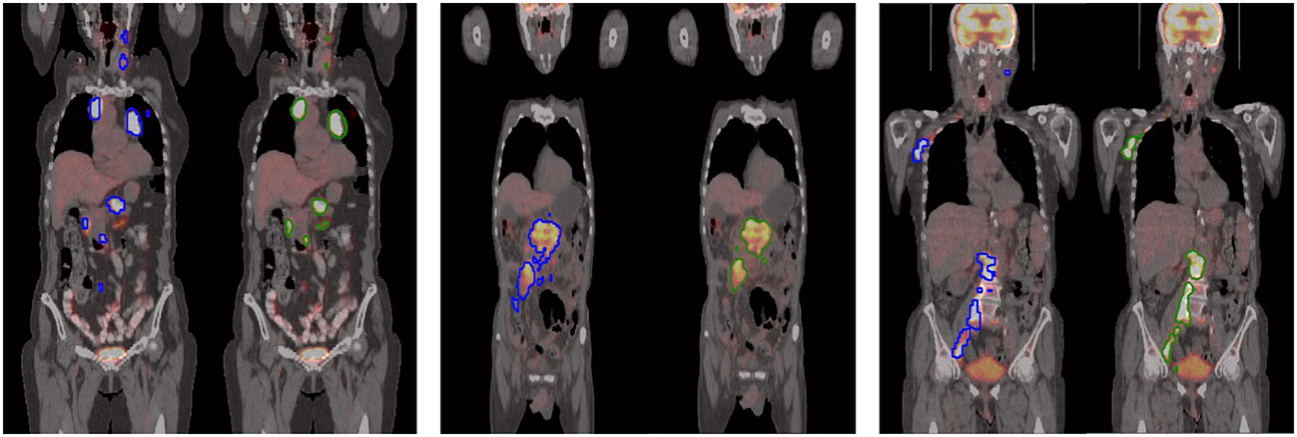
Eyes to thighs FDG-PET/CT fused coronal images from three different patient scans, showing ground truth ROIs in blue (left subpanel) and model predicted ROIs in green (right subpanel)

Segmentation results are presented in Table 1 and Fig. 3. As illustrated by the examples in Fig. 3, the predicted masks (green) have good spatial agreement with the ground truth (blue), although there are examples where small lesions tend to be underestimated (e.g., Figure 3, neck lesions in Patient 1). Overall, this method produced a DSC of 0.886 (0.862 when only using the 2D masks, 0.873 when only using the 3D masks) on the FL test dataset and a voxel level sensitivity of 92.6% and 93.0% for each test set (cf. Table 1). This level of performance was obtained on eyes to thighs datasets where overall lesion burden is sparse and anatomical background is highly heterogeneous. Previous published work ([22, 23], with DSC of 0.732 and 0.85, respectively) was based on more limited, less sparse, and more homogeneous regional scans.

**Table 1:** Summary of eyes to thighs results on DLBCL, FL, and NSCLC datasets

Dataset	Number of scans	Dice score	Sensitivity
DLBCL (training)	2266	0.895	93.2
Follicular lymphoma (test)	1124	0.886	92.6
Lung cancer (test)	274	–	93.0

Only a partial “ground truth” is available for the NSCLC test set. Thus, only sensitivity is being reported for these scans

**Fig. 4:**
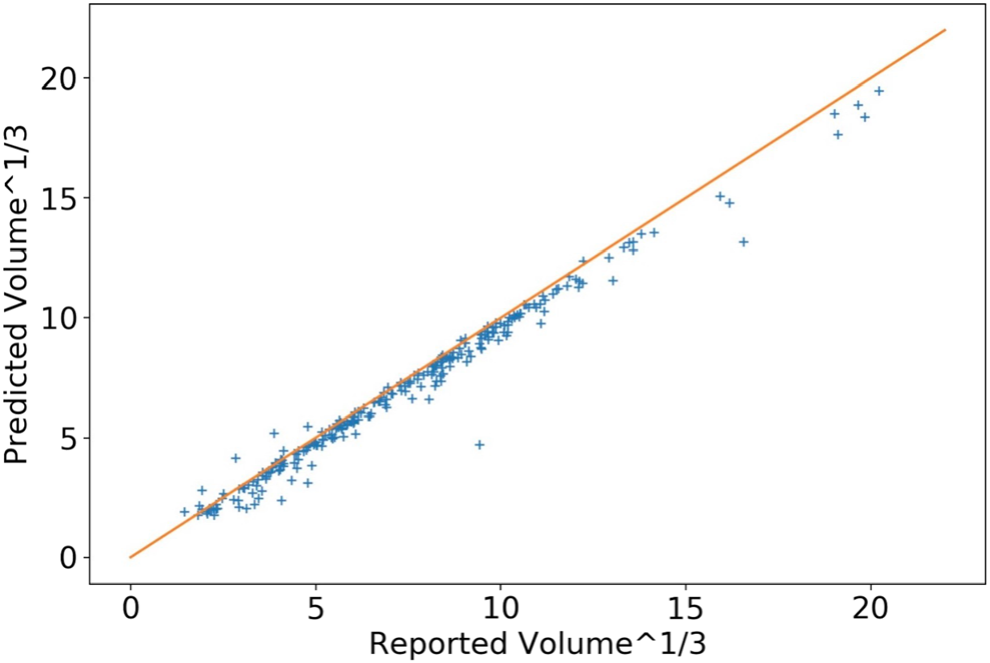
Comparison of automated total metabolic tumor volume with “ground truth” values in patients with FL

**Fig. 5:**
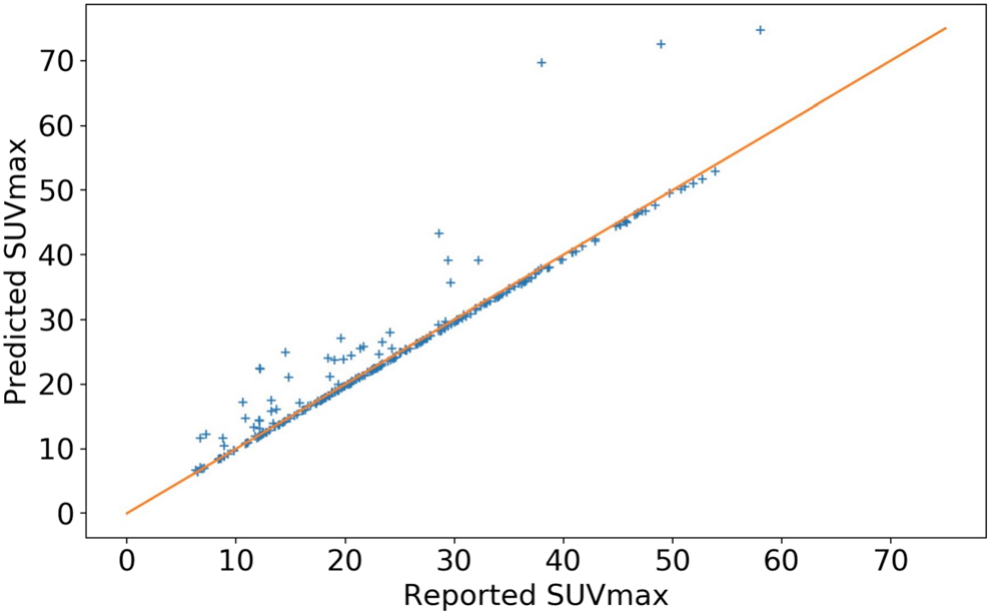
Comparison of automated SUV_max_ with “ground truth” values in patients with FL

Total metabolic tumor volume and SUV_max_ were calculated from the predicted tumor masks for each scan. As demonstrated in Fig. 4, the derivation of these metabolic tumor burden metrics yields very precise estimates compared with ground truth with Spearman’s correlations respectively of 0.97 and 0.96. This level of accuracy provides confidence that this novel, automated tool may be used to accurately and rapidly determine the burden of metabolically active disease in patients with solid tumors or lymphomas. The SUV_max_ correlation plot in Fig. 5 is performed at the patient level. A small fraction of the points does not lie close to the diagonal (90% of the predictions fall within 11.4% of the reported SUV_max_) and they all lie above the diagonal line. Possible explanations for these points fall into two general categories. Firstly, max-statistics are subject to large variability, and thus, reported SUV_max_ values could be underestimated, and SUV_max_ are very sensitive to noise in the predictions (overlap with physiological noise); in addition, artifacts in the image can cause ringing during resampling; this also elevates the SUV_max_ for correctly classified tumors.

## Conclusion

We present a novel memory-efficient NN architecture that enables a robust and rapid automated segmentation of tumors from 3D eyes to thighs FDG-PET/CT scans without need of downsampling. The automatic tumor segmentation showed strong agreement with radiologist’s segmentation used as ground truth (Table 1). The derived estimates of TMTV and SUV_max_ were highly correlated with the corresponding ground truth metrics (Fig. 4). Our experiments show that this model, trained solely on a large dataset of DLBCL patient scans, produces robust results in FL and NSCLC patient scans. These results are encouraging for the general application to other cancers, but specific application of this methodology to other cancer types will likely offer unique challenges associated with the specific cancer. Biological factors such as FDG avidity of a particular cancer type, common locations of metastatic disease, may require methodology differences in pre-processing, training, and post-processing. False positives that are likely physiological noise (e.g., misclassified heart or bladder uptake), where the SUV values were high, especially in patients with low tumor burden, may also need to be further investigated and addressed in future development. In future work, the model will be tested and adapted for scans acquired in other solid tumor cancer types, such as metastatic breast cancer and melanoma, and for longitudinal analysis. In addition, this architecture will be tested on other highly heterogeneous scans, such as diagnostic CT scans used for periodic tumor assessments in clinical trials.

The assessment of metabolic tumor burden by FDG PET has been found to be prognostic in many cancer types [3] and may be used to help inform and assess treatment decisions. Generally, total metabolic tumor burden is not measured in routine clinical practice, but having a fully automated methodology could provide radiologists and hematologists/oncologists with a rapid assessment of tumor burden which could inform risk stratification and potentially guide clinical patient management in the future. Our method demonstrates potential to provide radiologists with an automated, accurate, and rapid assessment of metabolic tumor burden in NHL and NSCLC patients. Future development is necessary to extend and validate this tool to other cancers and could provide radiologists with a valuable improvement to the radiologist workflow in assessing metabolic tumor burden.

## References

[1] Kello G et al: Progress and Promise of FDG-PET Imaging for Cancer Patient Management and Oncologic Drug Development. Clin Cancer Res 2005;11(8):2785–2808 10.1158/1078-0432.CCR-04-2626 15837727

[2] St-Pierre F, Broski SM, LaPlant BR, et al: Detection of extranodal and spleen involvement by FDG-PET imaging predicts adverse survival in untreated follicular lymphoma. Am J Hematol 2019;94:786–793. 10.1002/ajh.25493 10.1002/ajh.25493 31006875

[3] Chen HHW, Chiu N-T (2012). Prognostic Value of Whole-Body Total Lesion Glycolysis at Pretreatment FDG PET/CT in Non-Small Cell Lung Cancer. Radiology.

[4] Young H, et al: Measurement of clinical and subclinical tumor response using [18F]-fluorodeoxyglucose and positron emission tomography: review and 1999 EORTC recommendations. Eur J Cancer 1999;35(13):1773–1782 10.1016/s0959-8049(99)00229-4 10673991

[5] Cheson B, et al: Revised Response Criteria for Malignant Lymphoma. J Clin Oncol 2007;25(5):579–586 10.1200/JCO.2006.09.2403 17242396

[6] Cheson B (2014). Recommendations for Initial Evaluation, Staging, and Response Assessment of Hodgkin and Non-Hodgkin Lymphoma: The Lugano Classification. J Clin Oncol.

[7] Long J, Shelhamer E, Darrell T: Fully convolutional networks for semantic segmentation,CVPR. IEEE Computer Society, 2015, pp 3431–3440 10.1109/TPAMI.2016.2572683 27244717

[8] He K, Gkioxari G, Dollar P, Girshick P: Mask R-CNN, In: 2017 IEEE International Conference on Computer Vision. IEEE, 2017, pp 2980–2988. 10.1109/ICCV.2017.322

[9] Yan K, et al: DeepLesion: automated mining of large-scale lesion annotations and universal lesion detection with deep learning. J Med Imag 2018;5(3):1–11. 10.1117/1.JMI.5.3.036501 10.1117/1.JMI.5.3.036501 PMC6052252 30035154

[10] Kamnitsas K (2017). Efficient multi-scale 3D CNN with fully connected CRF for accurate brain lesion segmentation. Med Image Anal.

[11] Milletari F, Navab N, Ahmadi S: V-Net: Fully Convolutional Neural Networks for Volumetric Medical Image Segmentation, in Proc. Fourth International Conference on 3D Vision (3DV), 2016, pp 565–571. 10.1109/3DV.2016.79

[12] Ronneberger O, Fischer P, Brox T: U-Net: Convolutional Networks for Biomedical Image Segmentation. In: Navab N, Hornegger J, Wells WM, Frangi AF Eds. MICCAI 2015. LNCS, 9351, 2015, pp. 234–241. Springer, Cham. 10.1007/978-3-319-24574-428

[13] He K, Zhang X, Ren S, Sun J: Deep Residual Learning for Image Recognition, 2015. https://arxiv.org/abs/1512.03385

[14] Chollet F: Xception: Deep learning with depthwise separable convolutions. In The IEEE Conference on Computer Vision and Pattern Recognition (CVPR), 2017

[15] Yu F, Koltun V: Multi-Scale Context Aggregation by Dilated Convolutions, 2016. https://arxiv.org/abs/1511.07122

[16] Bauer C, Sun S, Sun W, et al. Automated measurement of uptake in cerebellum, liver, and aortic arch in full-body FDG PET/CT scans. Med Phys 2012;39(6):3112–23. 10.1118/1.4711815. 10.1118/1.4711815 PMC3365916 22755696

[17] Mathworks: Segment Lungs from 3-D Chest Scan and Calculate Lung Volume https://www.mathworks.com/help/images/segment-lungs-from-3-d-chest-mri-data.html. Accessed 25 Oct 2019

[18] Zou KH, Wareld SK, Bharatha A, et al: Statistical validation of image segmentation quality based on a spatial overlap index. Acad Radiol 2004;11(2):178–89. 10.1016/S1076-6332(03)00671-8 10.1016/S1076-6332(03)00671-8 PMC1415224 14974593

[19] Vitolo U, Trněný M, Belada D, Burke JM, Carella AM, Chua N, Abrisqueta P, Demeter J, Flinn I, Hong X, Kim WS, Pinto A, Shi YK, Tatsumi Y, Oestergaard MZ, Wenger M, Fingerle-Rowson G, Catalani O, Nielsen T, Martelli M, Sehn LH. Obinutuzumab or Rituximab Plus Cyclophosphamide, Doxorubicin, Vincristine, and Prednisone in Previously Untreated Diffuse Large B-Cell Lymphoma. J Clin Oncol 2017;35(31):3529–3537 10.1200/JCO.2017.73.3402 28796588

[20] Marcus R, Davies A, Ando K, Klapper W, Opat S, Owen C, Phillips E, Sangha R, Schlag R, Seymour JF, Townsend W, Trněný M, Wenger M, Fingerle-Rowson G, Rufibach K, Moore T, Herold M, Hiddemann W. Obinutuzumab for the First-Line Treatment of Follicular Lymphoma. N Engl J Med 2017;377(14):1331–1344 10.1056/NEJMoa1614598 28976863

[21] Kingma DP, Ba J: Adam: a method for stochastic optimization. In: 2015 Proceedings of the 3rd International Conference on Learning Representations (ICLR), 2015. Preprint at http://arxiv.org/abs/1412.6980

[22] Huang, B., Chen, Z., Wu, P.-M., et al: Fully Automated Delineation of Gross Tumor Volume for Head and Neck Cancer on PET-CT Using Deep Learning: A Dual-Center Study. Contrast Media Mol Imaging 2018. 10.1155/2018/8923028 10.1155/2018/8923028 PMC6220410 30473644

[23] Teramoto A, Fujita H, Yamamuro O, TamakiT: Automated detection of pulmonary nodules in PET/CT images: Ensemble false-positive reduction using a convolutional neural network technique. Med Phys 2015;49(6):2821–2827. 10.1118/1.4711815 10.1118/1.4948498 27277030

